# Kinnow madarin (*Citrus nobilis lour* × *Citrus deliciosa tenora*) fruit waste silage as potential feed for small ruminants

**DOI:** 10.14202/vetworld.2015.19-23

**Published:** 2015-01-06

**Authors:** B. A. Malla, A. Rastogi, R. K. Sharma, A. Ishfaq, and J. Farooq

**Affiliations:** 1 Department of Dairy Cattle Nutrition, National Dairy Research Institute, Karnal, Haryana, India; 2 Division of Animal Nutrition, Sher-e-Kashmir University of Agricultural Sciences & Technology of Jammu, Jammu, Jammu and Kashmir, India

**Keywords:** citrus waste, goats, kinnow mandarin waste, silage

## Abstract

**Aim:**

Study was conducted to ascertain the quality of Kinnow mandarin waste (KMW) silage and its utilization by adult male goats.

**Materials and Methods:**

KMW was collected, dried to 30% dry matter level and ensiled in silo pit after addition of disodium hydrogen orthophosphate as source of phosphorus as KMW is deficient in phosphorus. Oat was collected at milking stage, chopped finely and ensiled in a silo pit for 2 months. Twelve nondescript local adult male goats of about 8-10 months age and mean body weight of 23.00±0.90 kg were selected. The goats were randomly allotted on body weight as per randomized block design into two equal groups, six animals in each group (n=6) namely “oat silage (OS)” and “Kinnow silage.” Goats were offered weighed quantities of respective silage on *ad libitum* basis. The silages were evaluated for proximate principles and silage quality attributes.

**Results:**

Differences were found between chemical composition of both silages with higher organic matter, ether extracts, nitrogen free extract (p<0.05) and lower (p<0.01) crude fiber, neutral detergent fiber and acid detergent fibre concentration in KMW silage as compared to OS. However, silages were isonitrogenous (8.20 vs. 8.17; p>0.05 for CP) and possess comparable (2.23 vs. 2.06; p>0.05) calcium content. The pH, ammonia nitrogen (percent of total nitrogen) and soluble carbohydrate content were lower (4.20 vs. 3.30; 4.14 vs. 3.80; 2.73 vs. 1.86; p<0.05) in KMW silage, whereas, lactic acid concentration was higher (6.23 vs. 8.14; p<0.05) in KMW silage indicating its superior quality as compared to OS. Body weight (kg) of goats and silage intake (g/day), were comparable (p>0.05) among the two dietary groups.

**Conclusion:**

It can be concluded that KMW can be used to prepare good quality silage for feeding of goats.

## Introduction

In many parts of the world, the development of animal production is limited due to low rainfall and shortage of feed resources. India has a shortage of 32, 20 and 25% of concentrates, green forages and crop residues, respectively [1]. High feed costs, feed shortage and limited access to conventional feed ingredients have forced livestock breeders to use agricultural and food processing by-products as animal feed. Usage of such non-conventional feeds would not only decrease the demand for cereal grains used as animal feed, but also eliminates the need for developing costly waste management programs and may prevent problems from their disposal into the environment. The improper disposition of these by-products implies, logically, the environment deterioration. Such waste being moisture and nutrient rich gets easily putrefied and emits acidic effluents and smell. Moreover, these waste can deplete dissolved oxygen from contaminated water bodies via phenomenon of algal bloom. The disposal may appreciably increase environmental pollution due to its rapid decay, thus becoming a good source of house fly multiplication. Fruit and vegetable processing, packing, distribution and consumption generate a huge quantity of fruit and vegetable wastes (FVWs). For example, approximately 1.81, 6.53, 32.0 and 15.0 million tons of FVW are generated in India, the Philippines, China and the United States of America, respectively, and most of this is being disposed of either by composting or dumping in the landfills/rivers, causing environmental pollution [2].

“Kinnow,” a hybrid between king and willow mandarins (*Citrus nobilis* Lour × *Citrus deliciosa* Tenora) is one of the important citrus fruit crops in Northern Indian States [3]. Jammu and Kashmir produces 19070 MT of citrus fruit [4], majority of which is produced in Jammu Division and out of which a considerable share is that of Kinnow-mandarins. Kinnow peel and pulp are the by-products of the kinnow juice processing industry and account for about 55-60% of the fresh fruit weight [5]. After extraction of juice the remainder of the fruit, i.e., peel, membranes, juice vesicles and seed, are discarded as waste. About 30% of the production of citrus fruits (and 40% of orange production) is processed [6], principally to make juice. India ranks fourth in citrus fruit production and generates about 7.8 million tons of waste annually while world average is about 119.7 million tons [7,8].

The main advantage of dietary fiber from citrus fruits is its higher proportion of soluble dietary fiber when compared with other alternative sources such as cereals [9]. Moreover, citrus fruits have better quality than other sources of dietary fiber due to the presence of associated bioactive compounds (flavonoids and vitamin C) with antioxidant properties, which may provide additional health-promoting [10,11]. Foodborne pathogenic bacteria such as *Escherichia coli* O157:H7, *Salmonella typhimurium* are threats to the safety of ruminants. Citrus peel and dried orange pulp are by-products from citrus juice production that have natural antimicrobial effects. Orange pulp and/or peel included in ruminant diets decreases ruminal populations of these foodborne pathogenic bacteria and can be used as a preharvest intervention strategy as part of an integrated pathogen reduction scheme [11]. Due to the high moisture and sugar content, the huge load of mold and yeast, citrus peel is liable to rapid deterioration [12]. Therefore, preservation must be considered. Drying fresh by-products is an expensive procedure. Heat treatment volatilises citrus essential oils and hence there might not be enough citrus essential oils remaining to inhibit aflatoxins and harmful microbes [13]. Preservation of various types of organic byproducts by anaerobic fermentation represents a simple, low-cost alternative that can be conducted by the farmer.

This study was intended to evaluate Kinnow Mandarin waste (KMW) silage for goats.

## Materials and Methods

### Ethical approval

The experiment followed the guidelines of Institutional Animal Ethics Committee.

### Preparation and chemical evaluation of silage

KMW was collected from local juice vendors dried to achieve 30% dry matter (DM) level and then ensiled in a concrete pit silo for 2 months. To cater for skewed calcium: phosphorus ratio of KMW, as reported by Kour [14], disodium hydrogen orthophosphate was added to KMW while ensiling at the rate of 2.5 g/kg. Citrus by-products including dried citrus pulp have unbalanced Ca:P ratio that may cause milk fever in dairy cows [15]. Oat fodder was collected from local farmers at milking stage, chopped and wilted to 30% DM and ensiled in a similar silo pit as described above for 2 months. The silages were analyzed for proximate principles and calcium, phosphorus [16], fiber fractions [17], soluble sugars [18], lactic acid [19], ammonia nitrogen concentration [20], DM estimation by toluene distillation [16].

### Feeding animals

Twelve local nondescript adult male goats of about 8-10 months age and mean body weight of 23.00±0.90 kg were selected after approval by Institutional Animals Ethics Committee. Goats were randomly allotted on body weight as per randomized block design into two equal groups (n=6) namely “Oat silage (OS)” and “Kinnow silage (KS)” kept under uniform management conditions. *In vivo* feeding, trial was conducted for 30 days. Goats were adapted to the experimental diets for period of 1 week prior to feeding trial. Goats were individually offered weighed quantities of respective silage on *ad libitum* basis. The goats were fed twice daily, 08:00 and 17:00 h with free-access to water. Feed offered and refusals were recorded daily before the addition of fresh feed, and DM content was determined to calculate individual voluntary feed intakes (DM intake [DMI]). Body weight of each goat was recorded in the morning before feeding and watering at the onset of the feeding trial and then at weekly intervals throughout the experimental period.

### Statistical analysis

Data were analyzed using independent sample t-test for chemical composition; however, the body weight and intake data was subjected to general linear model multivariate analysis [21] and Means having significant differences were ranked [22]. The statistical analysis of experimental data was performed by using the statistical package SPSS statistical package SPSS (2005) version 16.0 Chicago, II, USA.

## Results and Discussion

### Chemical composition of feedstuffs

The proximate composition and fiber fractions (percent DM) of KMW silage and OS used in this study are detailed in Table-1.

**Table-1:** Chemical composition (%) of OS and KMW silage

Attributes*	OS	KMW silage	p value
Moisture	73.00±0.29	75.00±0.23	<0.05
OM	92.50±0.29	95.50±0.29	<0.05
CP	8.20±0.18	8.07±0.10	>0.05
EE	3.00±0.01	4.90±0.21	<0.01
CF	32.20±0.60	13.50±0.33	<0.01
Total ash	7.50±0.29	5.50±0.29	<0.01
Acid insoluble ash	1.53±0.05	1.00±0.29	>0.05
NFE	56.60±0.64	73.60±0.31	<0.01
NDE	53.00±0.93	33.00±0.58	<0.01
ADF	33.00±0.29	22.00±0.35	<0.01
Calcium	2.23±0.09	2.06±0.06	>0.05
Phosphorus	1.21±0.06	0.95±0.01	<0.05

* All values except moisture are expressed on DM basis and are mean±SE for analysis in triplicates. OM=Organic matter, CP=Crude protein, EE=Ether extract, CF=Crude fiber, NFE=Nitrogen free extract, NDE=Neutral detergent fiber, ADF=Acid detergent fiber, OS=Oat silage, KMW=Kinnow mandarin waste, SE=Standard error

There were differences (p<0.01) between chemical composition of both silages with respect to organic matter (OM), ether extracts (EE), crude fibre (CF), nitrogen free extract (NFE), Neutral detergent fibre (NDF) and acid detergent fibre (ADF). The concentration of OM, EE and NFE was higher, but the concentration of CF, NDF and ADF was lower in KMW silage as compared to OS. However, silages were isonitrogenous (8.20 vs. 8.07; p>0.05 for CP) and possess comparable (2.06 vs. 2.23; p>0.05) calcium content, although phosphorus content was higher (1.21 vs. 0.95; p<0.05) in OS. The chemical composition of KMW silage is in accordance with results obtained for other citrus fruit silage as reported by others [23-27]. KMW silage was found to be marginal in protein (8.07%) and rich in NFE (73.6%) indicating its potential as energy supplement in ruminant ration. Further, it has been suggested that citrus fruit waste are rich in pectin, which has a favorable effect on rumen environment for cellulolysis [28]. Its optimum CP, EE, NFE and NDF content pitches it as a complete feed for adult goats. The CP content of both silages is sufficient to satisfy the CP requirement of goats as per [29]. The citrus fruit waste is generally low in phosphorus [14,15] and addition of disodium hydrogen orthophosphate during ensiling resulted in increased phosphorus content in KMW silage, thereby amending the inherent skewed calcium: phosphorus ratio in KMW [14].

The chemical composition of OS prepared in the present study is in accordance with that reported by various previous workers [30-33]. Oat is said to be a preferred fodder for ensiling that make it an obvious choice for pitching as control feed against KMW silage. The results generated by feeding OS can, therefore, be considered standard and thus can be used to verify the feeding value of an unconventional feedstuff like KMW silage.

### Silage quality attributes

The quality attributes of the silages prepared in the study are detailed in Table-2. Color of silage produced was green in case of oats and yellow in KS. No discoloration or black/brownish spots were observed. Odor of both the silage was pleasant with oats having sweet smell and KMW having fruity smell. The DM content was 27% in OS and 25% in KMW silage with a lower (4.14 vs. 3.80; p<0.01) pH and higher lactic acid (6.23 vs. 8.14; p<0.01) concentration in KMW silage as compared to oats silage. The concentration of ammonia nitrogen (per cent of total nitrogen) and residual water soluble carbohydrate content (%) was lower (4.20 vs. 3.30; 2.73 vs. 1.86; p<0.05) in KMW silage as compared to that in OS.

**Table-2:** Quality characteristics of OS and KMW silage.

Attributes*	OS	KS	p value
Color	Green	Yellow	
Odor	Lactic acid	Lactic acid	
pH	4.14±0.06	3.80±0.02	<0.01
DM (%)	27.00±0.28	25.0±0.23	<0.01
Lactic acid (% DM)	6.23±1.08	8.14±1.12	<0.01
NH3 N (% total N)	4.20±0.30	3.30±0.45	<0.05
Water soluble carbohydrates (% DM)	2.73±0.35	1.86±0.30	<0.05

* All values except color and odor are mean±SE for analysis in triplicates. DM=Dry matter, OS=Oat silage, KMW=Kinnow mandarin waste, SE=Standard error, KS=Kinnow silage

No discoloration and foul odor was observed in both oats and KMW silage, indicating no aerobic putrefaction and carotene loss. The pH was lower (p<0.01) in KS as compared to OS showing high fermentation quality of KS. This is in agreement to previous reports that suggested high ensilability of citrus pulp due to the high concentration of rapidly fermentable carbohydrates [34,35]. This was also confirmed by the observation of higher (p<0.01) lactic acid concentration in KMW silage. Lower (p<0.05) concentration of ammonia nitrogen in KMW silage as compared to oats silage indicates lower protein degradation in KMW silage. This is in line to the lower pH and higher lactic acid concentration in KMW silage. Values of all these variables suggest that the fermentation was rapid in KMW silage. Lower (p<0.05) residual water-soluble carbohydrate content in KMW silage indicates that the fermentation was complete at the time of opening silo.

### Feed intake and live body weight

The weekly mean feed intake and live body weight of experimental goats during feeding trial are given in Tables-3 and 4, respectively. Total feed intake (on DM basis) was 554.33±3.55 g/day for the control group fed OS and 563.72±3.67 g/day for the treatment group fed with KMW silage. Silage intake (g/day) was comparable (p>0.05) between both the experimental groups. Intake was also similar (p>0.05) across different weeks throughout the feeding trial. Body weight of the goats did not vary between periods (p>0.05) and between dietary treatments (p>0.05).

**Table-3:** Weekly DM intake (g/day) of goats during the feeding trial.

Attributes	Week				Group mean±SEM	p value		
	
	I	II	III	IV		Group	Period	G×P*
OS	551.79±6.16	548.69±8.62	555.53±9.31	561.33±3.87	554.33±3.55	>0.05	>0.05	>0.05
KS	559.34±10.40	557.87±7.55	564.80±4.76	572.88±5.66	563.72±3.67			
Period mean±SEM	555.56±5.88	553.26±5.63	560.17±5.17	567.11±3.70	559.02±2.61			

* Group×period interaction, DM=Dry matter, OS=Oat silage, KS=Kinnow silage, SEM=Standard error mean

**Table-4:** Periodic live weight (kg) of goats during feeding trial.

Attributes/treatments	Days from onset of feeding trial (period)					Group mean±SEM	p value		
	
	0 day	8^th^ day	15^th^ day	22^nd^ day	30^th^ day		Group	Period	G×P*
OS	22.56±2.44	21.92±2.24	23.32±2.13	22.02±2.25	22.78±2.10	22.52±2.14	>0.05	>0.05	>0.05
KS	22.90±1.91	22.61±2.75	23.26±1.26	23.43±1.51	23.50±1.89	23.14±1.83			
Period mean±SEM	22.73±2.10	22.27±2.42	23.29±1.67	22.72±1.97	23.14±1.94	22.83±2.00			

* Group×period interaction, OS=Oat silage, KS=Kinnow silage, SEM=Standard error mean

Citrus pulp has little effect over the palatability of the ration, except for its distinctive smell. Citrus pulp should be introduced gradually into a ration to allow the animals enough time to become accustomed to its distinctive smell and taste [36]. In the present study, where after a short duration of about a week of gradual introduction of KMW silage in the ration of KS group animals, prior to start of feeding trial, they got accustomed to its distinctive taste and smell, hence the feed intake remained unaffected throughout the feeding trial. Mean daily DMI was comparable (p>0.05) between the OS and KS groups, indicating the similar acceptability of both silages by the experimental animals. All the experimental animals maintained their body weight throughout the feeding trial. This indicates that the nutrient supply from KMW and OS was sufficient for satisfying maintenance requirement of adult male goats. This is in accordance with the observations of other studies employing citrus fruit waste silage [37,38].

## Conclusion

The KMW makes good quality silage, which can effectively provide the maintenance requirements of small ruminants. Ensiling of same can reduce the potential threat of KMWs as an environment pollutant and also provides kinnow mandarin silage as a non-conventional feed for small ruminants.

## Authors’ Contributions

BAM carried out the research work and drafted the manuscript, AR and RKS planned and supervised the research work, AI and JF helped in conducting animal trial and BAM and AR revised the manuscript. All authors read and approved the final manuscript.
